# Three New Cytotoxic Steroidal Glycosides Isolated from *Conus pulicarius* Collected in Kosrae, Micronesia

**DOI:** 10.3390/md15120379

**Published:** 2017-12-04

**Authors:** Yeon-Ju Lee, Saem Han, Su Hyun Kim, Hyi-Seung Lee, Hee Jae Shin, Jong Seok Lee, Jihoon Lee

**Affiliations:** 1 Marine Nautral Product Chemistry Laboratory, Korea Institute of Ocean Science and Technology, Ansan 15627, Korea; gkstoa1126@naver.com (S.H.); tngus173@kiost.ac.kr (S.H.K.); hslee@kiost.ac.kr (H.-S.L.); shinhj@kiost.ac.kr (H.J.S.); jslee@kiost.ac.kr (J.S.L.); jihoonlee@kiost.ac.kr (J.L.); 2 Department of Marine Biotechnology, University of Science and Technology, Daejeon 34113, Korea

**Keywords:** *Conus pulicarius*, steroidal glycoside, cholesterol sulfate, cytotoxicity, leukemia

## Abstract

Three new sulfated steroidal glycosides (**3**–**5**), along with known cholesterol derivatives (**1**,**2**), were isolated from the visceral extract of the cone snail *Conus pulicarius*. The structure of each new compound was elucidated by nuclear magnetic resonance spectroscopy and high-resolution mass spectrometry. The three new compounds exhibited significant in vitro cytotoxicity (GI_50_ values down to 0.49 μM) against the K562 human leukemia cell line.

## 1. Introduction

Cone snails are predatory marine molluscs that secrete venom to capture prey, such as marine worms, small fish, molluscs, and other cone snails. The venom that produced by cone snails is composed of various modified peptides, such as conotoxin, which is produced by the Conus species. Conotoxins have generated tremendous interest in the fields of biology and pharmacology, as these peptides cause neurophysiological responses by modulating the activity of ion channels. Over 100,000 neuroactive conotoxins have been discovered from cone snails, which now comprise over 1000 species in the tropical and subtropical areas [1], since the first conotoxins were isolated and characterized [2,3]. During these investigations, ziconotide, the conotoxin peptide that is derived from *Conus magus*, has gained approval from the Food and Drugs Administration (FDA) as an analgesic for severe and chronic pain, and several other conotoxins are now under investigation in clinical trials as medical agents for the same purpose [4,5].

**Figure 1:**
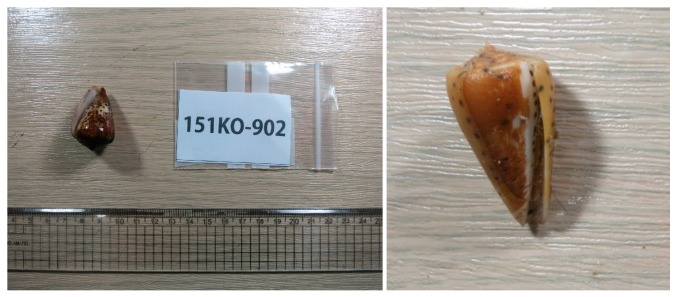
Specimen of *Conus pulicarius* collected in Kosrae.

**Figure 2:**
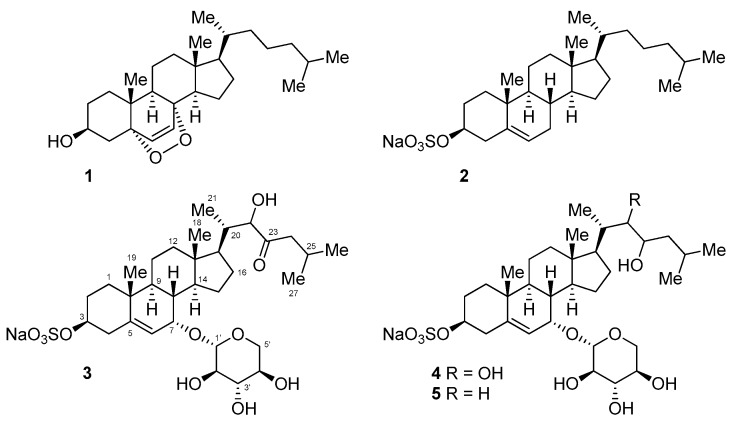
Structures of the isolated cholesterol derivatives.

Small molecules that are produced by the Conus species have gained much less attention as compared to conotoxins, presumably based upon the assumption that the predatory or protective mechanism of the Conus species is mainly dependent on the production and secretion of conotoxins. Despite a thorough literature search, only two publications on the small molecule metabolite that is isolated from the Conus species could be found. One publication describes the isolation of cholesterol and the epidioxysterol **1** from the three species of Conus cone snails (*Conus ebraeus*, *C. leopardus*, and *C. tessulatus*) [6], and the other describes the isolation of a guanine derivative that causes paralysis in mice [7]. Additionally, there is one publication that reported the isolation of thiazoline metabolites (pulicatins) from the cultivation of bacteria *Streptomyces* sp. CP32 associated with *Conus pulicarius* [8]. These findings led us to investigate the metabolites that contained in the Conus species, as marine invertebrates are considered to have diverse and complex symbiotic relationships and chemical defense mechanisms. In this work, we investigated the visceral extract of *Conus pulicarius* collected off the coast of Kosrae, the Federated States of Micronesia (Figure 1), in search for novel toxic metabolites. Herein, five cholesterol derivatives (**1**–**5**, Figure 2), which were isolated from this organism, have been described. While compounds **1** and **2** were the previously identified epidioxysterol and cholesterol sulfate, respectively, compounds **3**–**5** were new steroidal glycosides. In vitro cytotoxicity tests revealed that these new steroidal glycosides were extremely cytotoxic against the human leukemia cell line K562.

## 2. Results

### 2.1. Isolation of Cholesterol Derivatives from the Conus Pulicarius Cone Snails

The grinded and lyophilized whole-body tissue of *Conus pulicarius* specimens were extracted with methanol and dichloromethane. The combined extracts were partitioned between *n*-butanol and water, and the *n*-butanol fraction was subsequently partitioned between 15% aqueous methanol and *n*-hexane. The 15% aqueous methanol fraction was subjected to reverse-phase flash column chromatography using the ODS resin with aqueous methanol gradient elution (50% aqueous methanol to 100% methanol) to afford six fractions. The growth inhibitory activity of each fraction against the human leukemia cell line K562 was evaluated to trace the cytotoxic metabolites in the extract. The second and third fractions eluted with 40% (GI_50_ 3.4 μg/mL) and 30% aqueous methanol (GI_50_ 0.9 μg/mL), respectively, demonstrated high levels of cytotoxicity. These fractions were further purified by size-exclusion column chromatography on the Sephadex LH-20 column, and reverse-phase HPLC, using the YMC-Pack Pro C18 ODS column. Compounds **3** and **4** were obtained from the 40% aqueous methanol fraction, and **5** was obtained from the 30% aqueous methanol fraction. Known compounds **1** and **2** were obtained from the fractions that did not exhibit cytotoxicity at the concentration of 100 μg/mL (100% methanol and 10% aqueous methanol fractions, respectively).

### 2.2. Structure Elucidation of the Isolated Compounds

A comparison of nuclear magnetic resonance (NMR), mass spectrometry (MS), and optical rotation data with those reported previously confirmed that compounds **1** and **2** were 5α,8α-epidioxysterol and cholesterol sulfate [6,9,10,11] (Figure 2).

Compounds **3**–**5** were found to share the same tetracyclic carbon framework, with the only difference between these compounds being the substitution at C-17, as judged by ^1^H and ^13^C NMR (Table 1) and high-resolution mass spectrometry data.

**Figure 3:**
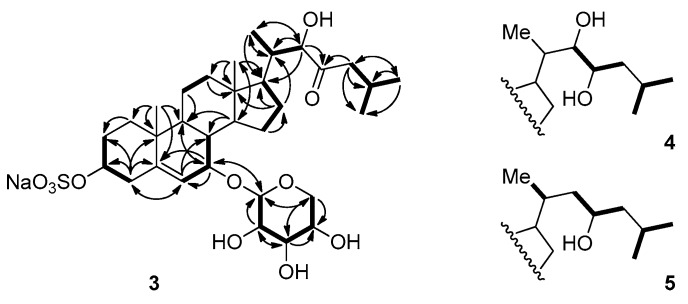
Selected COSY and HMBC correlations for compounds **3**–**5**.

Compound **3** was obtained as a colorless oil. Its molecular formula was determined as C_32_H_51_NaO_11_S by HRFABMS and ESIMS, which showed pseudomolecular ion peaks that were corresponding to [M − Na]^−^ and [M + Na]^+^, respectively. The tetracyclic carbon framework was analogous to that of cholesterol sulfate **2**, except for the presence of the oxymethine group at the C-7 position (δ_H_ 3.98, δ_C_ 70.1), as judged by the COSY correlation between the proton NMR signals at δ_H_ 5.74 (H-6), and 3.98 (H-7), in addition to the HMBC correlations between H-7 and the carbon signals at δ_C_ 148.2 (C-5), 122.0 (C-6), and 43.0 (C-9) (Table 1, Figure 3).

**Figure 4:**
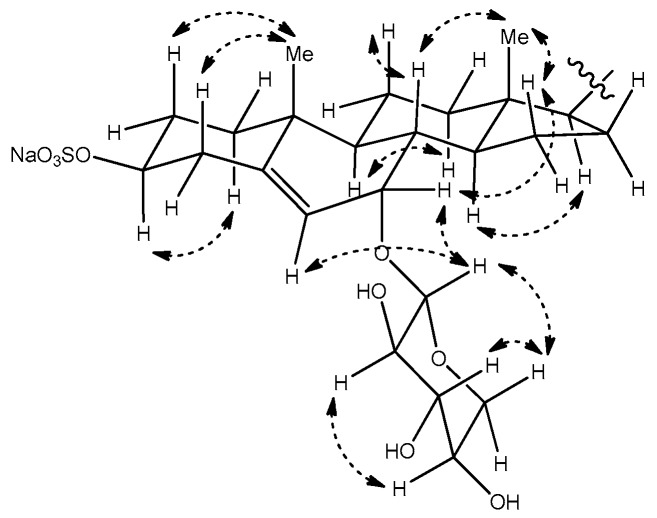
Selected NOESY correlations for compound **3**.

The relative configuration of the tetracyclic core was confirmed on the basis of coupling constants and correlations observed in the NOESY spectrum (Figure 4). Especially, the NOESY correlation between the proton signals at δ_H_ 4.21 (H-3) and 1.23 (H-1α), and the trans diaxial coupling (*J* = 13.0 Hz) of H-3 with an axial proton at C-4 (H-4β, δ 2.40), both supported the α orientation of H-3. The relative stereochemistry at C-7, where the additional oxygen is attached, was confirmed by a NOESY correlation between H-7 and H-15β (δ 1.10), which suggested that H-7 had the β-pseudoequatorial orientation. This assignment is in accordance with the previous reports, which describe compounds with similar structures as that of **1**. In the case of 3β,7β-dihydroxy-5-chole-24-oic acid, which was sulfated at C-3 and *N*-acetylglucosaminidated at C-7, the H-7 of the α-orientation appeared at δ 3.80 ppm, with a coupling constant of 7.8 to 8.4 Hz [12]. In other reports, comparative data are provided for the C-7 epimers of the synthesized 5-androstene-3,7,17-triol derivative. In the NMR spectra, the H-7 of the 7β-derivative oriented to the α-face appeared as a doublet with *J* = 7.9 Hz, whereas that of the 7α-derivative appeared as a broad singlet [13]. The same tendency has been observed in the case of 24-methylene- cholest-5-ene-3β,7α-diol and its C-7 epimer [14]. As the H-7 of compound **3** appeared as a broad singlet, this proton was confirmed to have an β-orientation.

The following proton signals were common in the ^1^H NMR spectra of all three compounds—a dioxymethine signal at δ 4.40 (H-1′), three hydroxymethine signals at δ 3.10 (H-2′), 3.31 (H-3′), and 3.46 (H-4′), and the signals of the two protons that were attached to the same oxymethylene carbon (C-5′, δ_C_ 66.8) at δ 3.83 (H-5′α) and 3.19 (H-5′β). This consistency in signals indicated the presence of the sugar moiety, which was identified as β-d-xylose based upon the interpretation of the coupling constants and NOESY correlations between the signals in this region. In detail, a proton signal at the anomeric position (H-1′) was coupled to a signal of H-2′ with *J* = 7.4 Hz, and H-2′ was again coupled to H-3′ with *J* = 8.9 Hz. NOESY correlations were also observed between H-1′, H-3′, and H-5′β (Figure 3 and Figure 4). These observations hinted at a diaxial relationship existing between these protons, and thus the sugar moiety was identified as β-d-xylose. This assignment was in accordance with the result that was obtained by the treatment of compound **3** with 2N HCl, which showed that the ^1^H NMR data and optical rotation value of the hydrolysis product coincided with those of d-xylose. The xylose moiety was connected to C-7 of the tetracyclic carbon framework, as judged by the HMBC correlations between the anomeric proton and the C-7 oxymethine carbon.

The remaining oxymethine proton signal appearing at δ_H_ 4.12 (H-22) showed HMBC correlations with the carbon signals at δ_C_ 53.3 (C-17), 40.1 (C-20), and 13.3 (C-21), and the signal of the ketone carbon at δ_C_ 215.0 (C-23). The two signals of the protons attached to the α-carbon with the chemical shift of δ_C_ 48.5 (C-24) appeared at δ_H_ 2.38 and 1.96 (H-24), and these signals showed COSY and HMBC correlations with the proton and carbon signals of isopropyl methine (C-25, δ_C_ 25.4). Based on these observations, the side branch attached at C-17 could be established, as depicted in Figure 2 and Figure 3.

Compound **4** was obtained as a pale-yellow amorphous solid. The ^1^H NMR data for **4** was quite similar to that of **3**, except for the additional oxymethine proton signal at δ_H_ 3.55 (H-23). In the ^13^C NMR spectra, the corresponding carbon signal at δ_C_ 72.2 (C-23) was observed and the signal corresponding to the carbonyl carbon was absent. Based upon these observations, **4** was identified as the 22,23-dihydroxy derivative, i.e., the reduced form of **3**. This assignment is in accordance with the molecular formula of C_32_H_53_NaO_11_S that were derived from (–)-HRFABMS, the COSY, and HMBC correlations (Figure 3).

Compound **5** had one less oxymethine signal in its ^1^H and ^13^C NMR spectra as compared to **2**. Instead, a carbon signal was observed at δ_C_ 46.3 (C-22), which was correlated to the two protons that appeared at δ_H_ 1.44 and 1.54 (H-22) in the HSQC spectra. The molecular formula of C_32_H_53_NaO_10_S obtained by (–)-HRFABMS also suggests that **5** has one less hydroxy group as compared to **4**. Consequently, the structure of **5** established from the COSY and HMBC correlations was as depicted in Figure 2 and Figure 3.

For the establishment of absolute stereochemistry at C-22 of **3**, C-22 and C-23 of **4**, and C-23 of **5**, we have tried to prepare the MTPA ester derivatives of the aglycon. Disappointingly, attempts to obtain the desulfated aglycon under various conditions, such as using an acid (2N HCl, *p*-toluenesulfonic acid) or a base (pyridine, potassium carbonate) only resulted in the decomposition of the starting material with the formation of products whose ^1^H NMR spectrum had few assignable signals. The direct acylation of the obtained compounds using MTPA chloride and the acetonide formation of **2** also failed because of the decomposition of the starting material. No valid method for establishing the absolute configuration of the oxymethine stereogenic center of the side branch in the new compounds could be found up to now.

The relative stereochemistry of C-20 in compounds **3**–**5** was established to be the same as that of cholesterol sulfate **2** based on the comparison of ^1^H and ^13^C NMR data of **2** and **5**. In detail, the carbon signal of C-17 appeared at δ_C_ 58.2, and the signal of the attached proton appeared at δ_H_ 1.15 in the NMR spectra of **5**, whereas these signals were observed at δ_C_ 57.5 and δ_H_ 1.09 in the spectra of **2**. The signals of C-20, C-21 and the attached proton were also similar; δ_c-20_ 37.1, δ_c-21_ 19.2, and δ_H__-21_ 1.40, δ_H__-21_ 0.95 in the NMR data of **2**, δ_c-20_ 35.2, δ_c-21_ 20.0, and δ_H__-20_ 1.44, δ_H__-21_ 0.98 in those of **5** (see Supplementary Materials).

All of the isolated compounds were tested for in vitro cytotoxicity against the human leukemia cell line K562. While compounds **1** and **2** did not exhibit any cytotoxicity (GI_50_ > 60.00 μM), the new sulfated steroidal glycosides **3**–**5** demonstrated potent cytotoxicities with GI_50_ values of 1.50 ± 0.25 μM, 1.39 ± 0.05 μM, and 0.49 ± 0.03 μM, respectively. Staurosporine, used as a positive control, showed an GI_50_ value of 2.29 ± 0.02 μM with in the same 96 well-plate.

## 3. Discussion

Five cholesterol derivatives (**1**–**5**), including three new sulfated steroidal glycosides (**3**–**5**), were isolated from the *Conus pulicarius* that were collected in Kosrae, Micronesia. The characteristic structural features of the new compounds include the sulfate group at the C-3 position and the xylose linked to C-7, which is different from those of known steroidal glycosides. Sulfated steroidal glycosides have been primarily isolated from marine organisms, such as algae and invertebrates [15,16]. Especially, the starfishes of various species are frequently found to contain sulfated steroidal glycosides, called asterosaponins, which have a sulfate group at the C-3 position and various sugar moieties linked to C-6. Compounds (**3**–**5**) were named as Conusaponin A-C, as these are the first example of steroidal glycosides isolated from Conus species.

The new compounds showed potent growth inhibitory activity against the human leukemia cell line K562. This finding, combined with those regarding the previously reported steroidal glycosides with potent activities against various cancer cell lines [16,17,18,19,20,21,22], would provide new insights into the structure-activity relationships of cytotoxic sulfated steroidal glycosides.

## 4. Materials and Methods

### 4.1. General Procedures

The optical rotations were measured using a JASCO digital polarimeter in a 5 cm long cell. Fourier transform infra-red (FTIR) spectra were recorded on a JASCO FT/IR-4100 spectrometer (JASCO, Tokyo, Japan). ^1^H and ^13^C NMR spectra were recorded on Varian Unity 500 500 MHz and 125 MHz spectrometers, respectively. The chemical shifts have been reported in ppm and referenced to the solvent resonances, resulting from incomplete deuteration as the internal references (CD_3_OD: δ_H_ 3.31 ppm, δ_C_ 49.00 ppm). HPLC was performed with YMC-Pack Pro C18 columns using a Shodex RI-101 detector (Shoko Science, Yokohama, Japan).

### 4.2. Biological Material Collection, Extraction, and Isolation

Twenty specimens of *Conus pulicarius* (3–4 cm) were collected by hand at 1–3 m depth offshore of Kosrae, the Federated States of Micronesia, in January, 2015. The collected specimens (300 g, wet wt.) were immediately freeze-dried and kept at −20 °C until the time of our investigation. The specimens were thawed at room temperature in a fume hood for 3 h, and then the shell was removed from the viscera. The viscera were then grinded in a blender and lyophilized to yield the 17.7 g of a sticky solid. This solid was extracted using methanol (300 mL × 2) and dichloromethane (300 mL × 1) at room temperature. The combined extract (2.8 g) was partitioned between *n*-butanol and water, and the organic layer (820 mg) was further partitioned between 15% aqueous methanol and *n*-hexane. Subsequently, the aqueous methanol fraction (430 mg) was subjected to reverse-phase column chromatography (YMC Gel ODS-A, 60 Å, 230 mesh) with a stepped gradient solvent system of 50, 40, 30, 20, and 10% aqueous methanol, and 100% methanol. The fraction eluted with 40% aqueous methanol (34.0 mg) was then subjected to size-exclusion column chromatography (LH-20), followed by further purification by reverse-phase HPLC (YMC-Pack Pro C18) to afford **3** (3.2 mg) and **4** (5.0 mg). The 30% aqueous methanol fraction (59.0 mg) was also subjected to size-exclusion chromatography and reverse-phase HPLC to afford **5** (3.1 mg). The 10% aqueous methanol and 100% methanol fractions were purified by reverse-phase HPLC to afford **1** (2.8 mg) and **2** (0.8 mg), respectively.

Compound **3**: pale yellow amorphous solid; ${\left[\alpha \right]}_{D}^{25}$ −73.1 (*c* 0.5, CH_3_OH); UV λ_max_ (log ε) 285 (4.5), 211 (3.4) nm; IR (KBr) ν_max_ 3337, 2921, 2885, 1055, 1033, 1014 cm^−^^1^; ^1^H and ^13^C NMR (CD_3_OD, 500 and 125 MHz), see Table 1 and Supplementary Materials; (+)-LRESIMS *m/z* 689.84 [M + Na]^+^; (–)-HRFABMS *m*/*z* 643.3156 [M − Na]^−^ (calcd. for C_32_H_51_O_11_S, *m*/*z* 643.3152).

Compound **4**: pale yellow amorphous solid; ${\left[\alpha \right]}_{D}^{25}$ −59.1 (*c* 0.5, CH_3_OH); UV λ_max_ (log ε) 286 (3.2), 235 (3.5), 211 (4.5) nm; IR (KBr) ν_max_ 3726, 2866, 1055, 1032, 1012 cm^−^^1^; ^1^H and ^13^C NMR (CD_3_OD, 500 and 125 MHz), see Table 1 and Supplementary Materials; (+)-LRESIMS *m/z* 691.67 [M + Na]^+^; (–)-HRFABMS *m*/*z* 645.3307 [M − Na]^−^ (calcd. for C_32_H_53_O_11_S, *m*/*z* 645.3309).

Compound **5**: pale yellow amorphous solid; ${\left[\alpha \right]}_{D}^{25}$ −40.7 (*c* 0.5, CH_3_OH); UV λ_max_ (log ε) 277 (3.9), 236 (4.2), 211 (4.5) nm; IR (KBr) ν_max_ 3725, 2865, 1055, 1033, 1055 cm^−^^1^; ^1^H and ^13^C NMR (CD_3_OD, 500 and 125 MHz), see Table 1 and Supplementary Materials; (+)-LRESIMS *m/z* 675.70 [M + Na]^+^; (–)-HRFABMS *m*/*z* 629.3363 [M − Na]^−^ (calcd. for C_32_H_53_O_10_S, *m*/*z* 629.3359).

### 4.3. Cytotoxicity Assay

The growth inhibition assay against human leukemia cell line K562 was performed according to a published protocol [23,24]. In brief, the cells were added to a 96-well plate containing either a control (staurosprine) or the test compounds. After incubation for 48 h, 10 μL of the WST-1 solution was added to each well of the culture plate (containing 100 μL of the RPMI medium). After incubation for 2 h at 37 °C, the optical density (OD) of the assay solution was measured at 450 nm by using the ELISA plate reader. Cell viability was calculated as a percentage, with the following equation: % cell viability = (OD_sample_/OD_control_) × 100. The GI_50_ values were determined by plotting cell viability versus the concentration of compound. Results were reported as the average values and standard deviations of triplicate samples.

## Figures and Tables

**Table 1:** ^1^H and ^13^C NMR data (500 and 125 MHz) for compounds **3**–**5** ^a,b^.

Postion	3		4		5	
	δ_C_	δ_H_ (*J* in Hz)	δ_C_	δ_H_ (*J* in Hz)	δ_C_	δ_H_ (*J* in Hz)
1α	37.6	1.23 (m)	37.7	1.22 (m)	37.7	1.22 (m)
1β		1.88 (m)		1.86 (m)		1.88 (m)
2α	29.7	2.07 (br d, 12.9)	29.7	2.07 (br d, 12.0)	29.8	2.07 (br d, 12.4)
2β		1.65 (m)		1.65 (m)		1.64 (m)
3	79.3	4.21 (dddd, 16.0, 13.0, 4.8, 4.8)	79.3	4.20 (dddd, 15.0, 13.1, 5.5, 5.2)	79.3	4.20 (dddd, 16.0, 13.1, 4.5, 4.5)
4α	40.4	2.61 (dd, 13.0, 4.8)	40.4	2.61 (dd, 13.1, 5.2)	40.4	2.61 (dd, 13.1, 4.5)
4β		2.40 (dd, 13.0, 13.0)		2.42 (dd, 13.1, 13.1)		2.42 (dd, 13.1, 13.1)
5	148.2		148.1		148.2	
6	122.0	5.74 (dd, 5.5, 1.5)	122.1	5.74 (dd, 5.0, 1.3)	122.1	5.73 (dd, 4.9, 1.3)
7	70.1	3.98 (br s)	70.1	4.00 (br s)	70.2	3.96 (br s)
8	38.5	1.55 (ovl)	38.7	1.53 (ovl)	38.4	1.53 (ovl)
9	43.0	1.54 (ovl)	43.0	1.54 (ovl)	43.0	1.54 (ovl)
10	38.7		38.4		38.7	
11α	21.8	1.54 (ovl)	21.8	1.54 (ovl)	21.8	1.54 (ovl)
11β		1.21 (ovl)		1.29 (ovl)		1.29 (ovl)
12α	40.0	1.23 (ovl)	40.5	1.22 (ovl)	40.4	1.21 (ovl)
12β		1.93 (m)		1.98 (br d, 12.4)		2.00 (br d, 12.4)
13	43.0		43.0		43.3	
14	49.8	1.72 (m)	49.8	1.68 (m)	49.7	1.63 (m)
15α	24.6	1.97 (ovl)	24.7	1.95 (ovl)	24.8	1.92 (ovl)
15β		1.10 (m)		1.09 (m)		1.05 (m)
16α	29.0	1.98 (ovl)	28.9	1.95 (m)	29.6	2.05 (m)
16β		1.44 (m)		1.25 (ovl)		1.21 (ovl)
17	53.3	1.57 (ovl)	53.4	1.56 (ovl)	58.2	1.15 (m)
18	11.9	0.75 (s)	11.8	0.71 (s)	11.9	0.70 (s)
19	18.5	1.03 (s)	18.6	1.02 (s)	18.6	1.02 (s)
20	40.1	1.97 (m)	38.9	1.53 (m)	35.2	1.44 (m)
21	13.3	0.76 (d, 6.8)	12.6	0.91 (d 6.4)	20.0	0.98 (d, 6.4)
22	80.8	4.12 (br s)	78.2	3.31 (ovl)	46.3	1.44 (ovl)
						1.54 (ovl)
23	215.0		72.2	3.55 (ddd, 10.5, 8.2, 2.6)	69.1	3.69 (m)
24	48.5	2.38 (dd, 18.3, 6.8)	43.3	1.14 (ddd, 13.6, 10.5, 2.6)	47.5	1.22 (ovl)
		1.96 (ovl)		1.24 (ovl)		1.25 (ovl)
25	25.4	2.12 (m)	25.3	1.86 (m)	25.5	1.84 (m)
26	22.9	0.91 (d, 6.8)	21.7	0.92 (d, 6.6)	22.0	0.90 (d, 6.7)
27	23.0	0.93 (d, 6.8)	24.5	0.94(d, 6.7)	22.0	0.92 (d, 6.7)
1′	101.3	4.40 (d, 7.4)	101.3	4.40 (d, 7.4)	101.3	4.40 (d, 7.5)
2′	75.2	3.10 (dd, 8.9, 7.4)	75.2	3.10 (dd, 8.9, 7.4)	75.2	3.10 (dd, 8.9, 7.5)
3′	77.9	3.31 (ovl)	77.8	3.33 (ovl)	77.9	3.32 (ovl)
4′	71.4	3.46 (ddd, 10.0, 9.0, 5.3)	71.4	3.46 (ddd, 10.2, 9.4, 5.3)	71.4	3.47 (ddd, 9.9, 8.9, 5.3)
5′α	66.8	3.83 (dd, 11.4, 5.3)	66.8	3.83 (dd, 11.4, 5.3)	66.8	3.83 (dd, 11.4, 5.3)
5′β		3.19 (dd, 11.4, 10.0)		3.19 (dd, 11.4, 10.2)		3.19 (dd, 11.4, 9.9)

^a^ Data were obtained in CD_3_OD. ^b^ These assignments are based on HSQC, COSY, and HMBC results.

## Supplementary Materials

The following are available online at www.mdpi.com/1660-3397/15/12/379/s1, ^1^H and ^13^C NMR spectra of **1**–**5**, and 2D (HMBC, HSQC, COSY, NOESY) NMR spectra of **3**–**5**.
