# Supplementary material for: Three New Cytotoxic Steroidal Glycosides Isolated from Conus pulicarius Collected in Kosrae, Micronesia
Source: Mar Drugs. 2017 Dec 4;15(12):379. doi: 10.3390/md15120379 (PMC5742839; doi:10.3390/md15120379)

Supporting Informations

Three New Cytotoxic Steroidal Glycosides Isolated from *Conus pulicarius* Collected in Kosrae, Micronesia

Yeon-Ju Lee, * Saem Han, Su Hyun Kim, Hyi-Seung Lee, Hee Jae Shin, Jong Seok Lee, Jihoon Lee

Marine Natural Product Chemistry Laboratory, Korea Institute of Ocean Science and Technology, Ansan 426-744, Republic of Korea.

yjlee@kiost.ac.kr

Tables of Contents

Table S1. Complete NMR data of **3** in methanol-d_4

Table S2. Complete NMR data of **4** in methanol-d_4

Table S3. Complete NMR data of **5** in methanol-d_4

Figure S1. ^{1}H and ^{13}C NMR Spectra (500 MHz and 125 MHz, chloroform-*d*) of **1**

Figure S2. ^{1}H and ^{13}C NMR Spectra (500 MHz and 125 MHz, methanol-d_4) of **2**

Figure S3. ^{1}H NMR Spectra (500 MHz, DMSO-d_6) of **2**

Figure S4. ^{1}H and ^{13}C NMR Spectra (500 MHz and 125 MHz, methanol-d_4) of **3**

Figure S5. HMBC and HSQC NMR spectra of **3**

Figure S6. COSY and NOESY NMR spectra of **3**

Figure S7. ^{1}H and ^{13}C NMR Spectra (500 MHz and 125 MHz, methanol-d_4) of **4**

Figure S8. HMBC and HSQC NMR spectra of **4**

Figure S9. COSY and NOESY NMR spectra of **4**

Figure S10. ^{1}H and ^{13}C NMR Spectra (500 MHz and 125 MHz, methanol-d_4) of **5**

Figure S11. HMBC and HSQC NMR spectra of **5**

Figure S12. COSY and NOESY NMR spectra of **5**

Table S1. Complete NMR data of **3** in methanol-d_4

position	δ^{13}C	δ^{1}H (mult, *J* in Hz)	COSY	HMBC[c]	NOESY
1α	37.6	1.23 (m)	1β	2	2α , **3**
1β		1.88 (m)	1α		**19**
2α	29.7	2.07 (brd, 12.9)	2β		1α , 3
2β		1.65 (m)	2α		**4β**, **19**
3	79.3	4.21 (dddd, 16.0, 13.0, 4.8, 4.8)	4α, 4β		**1α**, 2α, 4α, 4β
4α	40.4	2.61 (dd, 13.0, 4.8)	3	2, 3, 5, 6, 10	3, **6**
4β		2.40 (dd, 13.0, 13.0)	3	3, 5, 6	**2β**, 3, **19**
5	148.2				
6	122.0	5.74 (dd, 5.5, 1.5)	7	7, 8	**4α**, 7, **1'**
7	70.1	3.98 (brs)	6	5, 6, 9, 1'	6, 8, **15α**, **1'**
8	38.5	1.55 (ovl)	14		7, **11β**, **18**
9	43.0	1.54 (ovl)			**12α**, **14**
10	38.7				
11α	21.8	1.54 (ovl)	11β		
11β		1.21 (ovl)	11α, 12β	9	**8**, **18**
12α	40.0	1.23 (ovl)	12β		**9**, **14**
12β		1.93 (m)	11β, 12α	13	
13	43.0				
14	49.8	1.72 (m)	8	8, 15	**12α**, **17**
15α	24.6	1.97 (ovl)	15β, 16β	16	
15β		1.10 (m)	15α		**7**, **1'**
16α	29.0	1.98 (ovl)	16β	13, 17, 20	
16β		1.44 (m)	15α, 16α, 17		
17	53.3	1.57 (ovl)	16β		**14**, 20, **22**
18	11.9	0.75 (s)		12, 13, 14, 17	**8**, **11β**
19	18.5	1.03 (s)		1, 5, 9, 10	**2β**, **4β**
20	40.1	1.97 (m)	21	21	22
21	13.3	0.76 (d, 6.8)	20	17, 20, 22	
22	80.8	4.12 (brs)		17, 20, 21, 23	17, 20
23	215.0				
24	48.5	2.38 (dd 18.3, 6.8)	25	23, 25, 26, 27	25
		1.96 (ovl)			
25	25.4	2.12 (m)	24, 26, 27	24	24
26	22.9	0.91 (d, 6.8)	25	24, 25, 27	
27	23.0	0.93 (d, 6.8)	25	24, 25, 26	
1'	101.3	4.40 (d, 7.4)	2'	7, 5'	**6**, **7**, **3'**, **5'β**
2'	75.2	3.10 (dd, 8.9, 7.4)	1', 3'	1', 3'	**4'**
3'	77.9	3.31 (ovl)	2', 4'	2', 4', 5'	**1'**, **5'β**

4'	71.4	3.46 (ddd, 10.0, 9.0, 5.3)	3', 5'	3', 5'	**2'**
5'α	66.8	3.83 (dd, 11.4, 5.3)	4'	1', 3', 4'	
5'β		3.19 (dd, 11.4, 10.0)	4'	1', 3', 4'	**1'**, **3'**

Table S2. Complete NMR data of **4** in methanol-d_4

position	δ^{13}C	δ^{1}H (mult, *J* in Hz)	COSY	HMBC^{c}	NOESY
1α	37.7	1.22 (m)	1β, 2β	2	2α, **3**
1β		1.86 (m)	1α		**19**
2α	29.7	2.07 (brd, 12.0)	2β, 1α		1α, 3
2β		1.65(m)	2α, 3		1α, **4β**, **19**
3	79.3	4.20 (dddd, 15.0, 13.1, 5.5, 5.2)	2β, 4α, 4β		**1α**, 2α, 4α
4α	40.4	2.61 (dd, 13.1, 5.2)	3	2, 3, 5, 6	3, **6**
4β		2.42 (dd, 13.1, 13.1)	3	3, 5	**2β**, **19**
5	148.1				
6	122.1	5.74 (dd, 5.0, 1.3)	7	8	**4α**, **1'**
7	70.1	4.00 (brs)	6, 8	5, 6	15α, **1'**
8	38.7	1.53 (ovl)	7, 14		**18**
9	43.0	1.54 (ovl)			**12α**, **14**
10	38.4				
11α	21.8	1.54 (ovl)	11β		
11β		1.29 (ovl)	11α, 12β	9	12β, **18**
12α	40.5	1.22 (ovl)	12β		**9**, **14**
12β		1.98 (brd, 12.4)	11β, 12α	13	18
13	43.0				
14	49.8	1.68 (m)	8	9	**9**, **12α**
15α	24.7	1.95 (ovl)	15β, 16β	13, 14	
15β		1.09 (m)	15α		**7, 1'**
16α	28.9	1.95 (m)	16β	20	
16β		1.25 (ovl)	15α, 16α		17, **22**
17	53.4	1.56 (ovl)			16β, **22**
18	11.8	0.71 (s)		12, 13, 14, 17	**8**, **11β**, 12β
19	18.6	1.02 (s)		1, 5, 9, 10	1β, **2β, 4β**
20	38.9	1.53 (m)	21		
21	12.6	0.91 (d 6.4)	20	17, 20, 22	23
22	78.2	3.31 (ovl)	23		16β**, 17**, 23
23	72.2	3.55 (ddd 10.5, 8.2, 2.6)	22, 24		21, 22
24	43.3	1.14 (ddd 13.6, 10.5, 2.6)	23, 25		
		1.24 (ovl)		23, 25, 26, 27	
25	25.3	1.86 (m)	24, 26, 27	24	
26	21.7	0.92 (d, 6.6)	25	24, 25, 27	
27	24.5	0.94(d, 6.7)	25	24, 25, 26	
1'	101.3	4.40 (d, 7.4)	2'	7	**6**, **7**, **3'**, **5'β**
2'	75.2	3.10 (dd, 8.9, 7.4	1', 3'	1', 3'	**3'**, 4'
3'	77.8	3.33 (ovl)	2', 4'	2'	**1'**, **5'β**

4'	71.4	3.46 (ddd, 10.2, 9.4, 5.3)	3', 5'		**2'**
5'α	66.8	3.83 (dd, 11.4, 5.3)	4'	1', 4'	**4'**
5'β		3.19 (dd, 11.4, 10.2)	4'	1', 3', 4'	**1'**, **3'**

Table S3. Complete NMR data of **5** in methanol-d_4

position	δ^{13}C	δ^{1}H (mult, J in Hz)	COSY	HMBC[c]	NOESY
1α	37.7	1.22(m)	1β, 2β	2	2β, **3**
1β		1.88 (m)	1α		19
2α	29.8	2.07 (brd, 12.4)	2β, 1α		1α, 2β, 3,
2β		1.64(m)	2α, 3		1α, **19**
3	79.3	4.20 (dddd, 16.0, 13.1, 4.5, 4.5)	2β, 4α, 4β		**1α**, 2α, 4α
4α	40.4	2.61 (dd, 13.1, 4.5)	3	3, 5	3, **6**
4β		2.42 (dd, 13.1, 13.1)	3	2, 3, 5, 6	3, **19**
5	148.2				
6	122.1	5.73 (dd, 4.9, 1.3)	7	8	**4α**, 7, 1'
7	70.2	3.96 (brs)	6, 8	5, 6	6, 1'
8	38.4	1.53 (ovl)	7, 14		**18**
9	43.0	1.54 (ovl)			**14**
10	38.7				
11α	21.8	1.54 (ovl)	11β		
11β		1.29 (ovl)	11α, 12β	9	**18, 19**
12α	40.4	1.21 (ovl)	12β		
12β		2.00 (brd, 12.4)	11β, 12α	13	18
13	43.3				
14	49.7	1.63 (m)	8	9	**9**, **17**
15α	24.8	1.92 (ovl)	15β, 16β	13, 14	**17**
15β		1.05 (m)	15α		**18**
16α	29.6	2.05 (m)	16β	20	
16β		1.21 (ovl)	15α, 16α		
17	58.2	1.15 (m)			**14, 15α**
18	11.9	0.70 (s)		12, 13, 14, 17	**8**, **11β**, 12β, **15β**
19	18.6	1.02 (s)		1, 5, 9, 10	1β, **2β, 4β, 11β**
20	35.2	1.44 (m)	21		
21	20.0	0.98 (d, 6.4)	20	17, 20, 22	
22	46.3	1.44 (ovl)	23		
		1.54 (ovl)		17, 20, 21, 23	23
23	69.1	3.69 (m)	22, 24		22 24
24	475	1.22 (ovl)	23, 25		23
		1.25 (ovl)		23, 25, 26, 27	
25	25.5	1.84 (m)	24, 26, 27	24	
26	22.0	0.90 (d, 6.7)	25	24, 25, 27	
27	24.3	0.92 (d, 6.7)	25	24, 25, 26	
1'	101.3	4.40 (d, 7.5)	2'	7	**6**, **7**, **3'**, **5'β**
2'	75.2	3.10 (dd, 8.9, 7.5)	1', 3'	1', 3'	3', **4'**

3'	77.9	3.32 (ovl)	2', 4'	2'	2', 1'
4'	71.4	3.47 (ddd, 9.9, 8.9, 5.3)	3', 5'		**2'**, 5'
5'α	66.8	3.83 (dd, 11.4, 5.3)	4'	1', 4'	4'
5'β		3.19 (dd, 11.4, 9.9)	4'	1', 3', 4'	**1'**

Figure S1. ^{1}H and ^{13}C NMR Spectra (500 MHz and 125 MHz, chloroform-*d*) of **1**

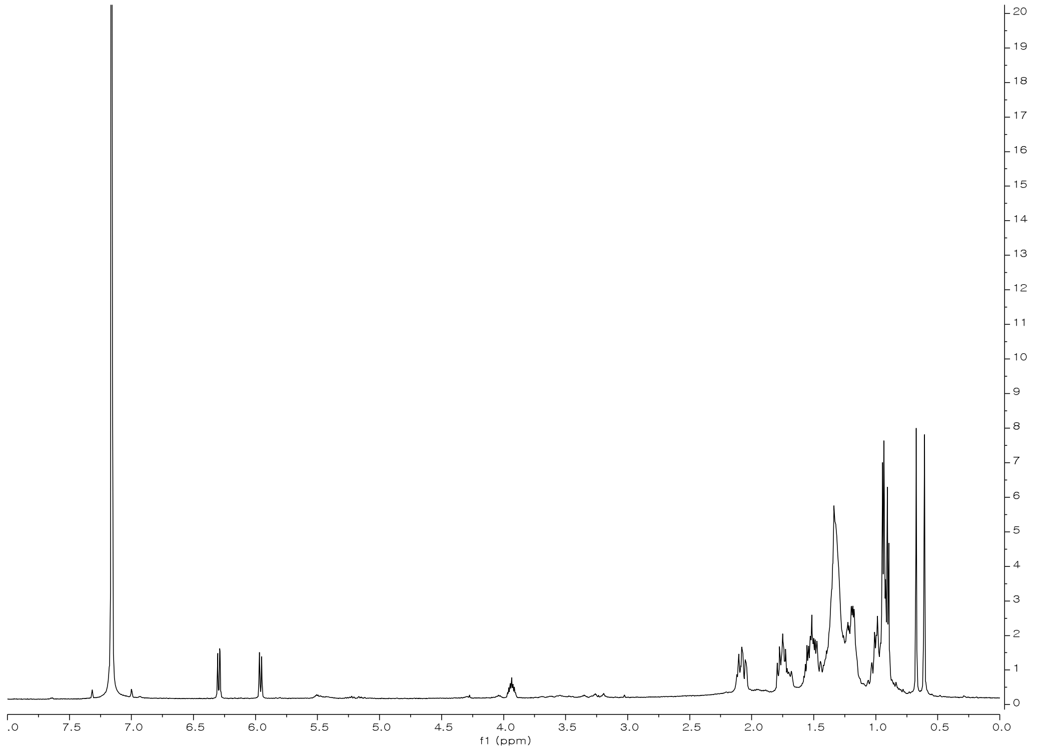

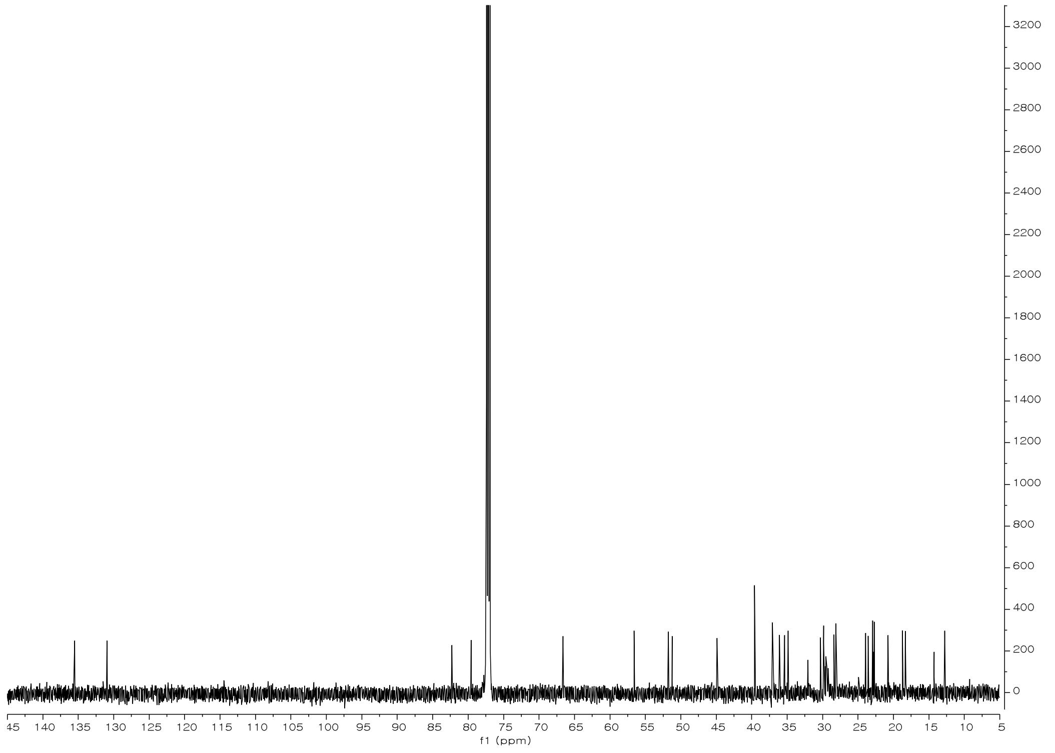

Figure S2. ^{1}H and ^{13}C NMR Spectra (500 MHz and 125 MHz, methanol-d_4) of **2**

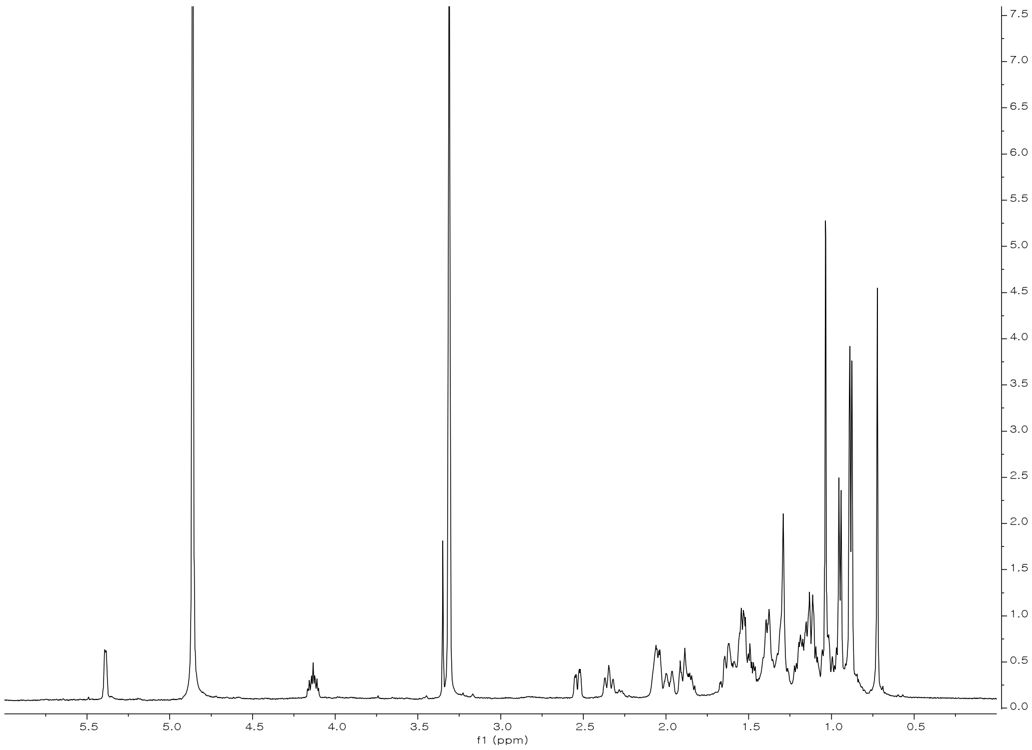

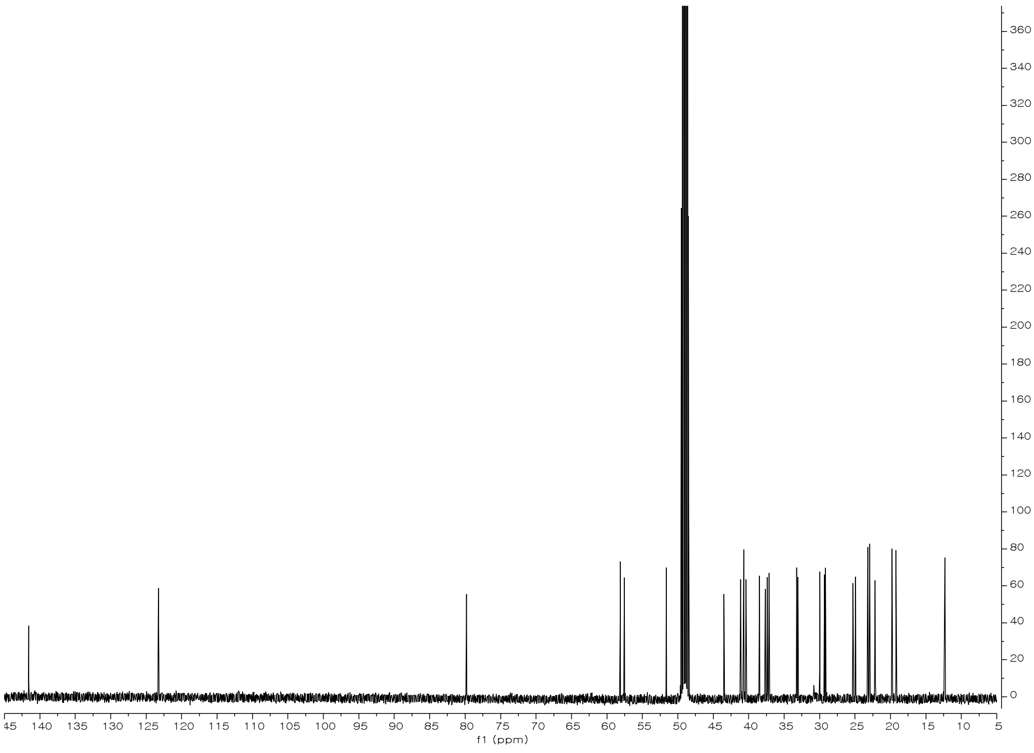

Figure S3. ^{1}H NMR Spectra (500 MHz, DMSO-d_6) of **2**

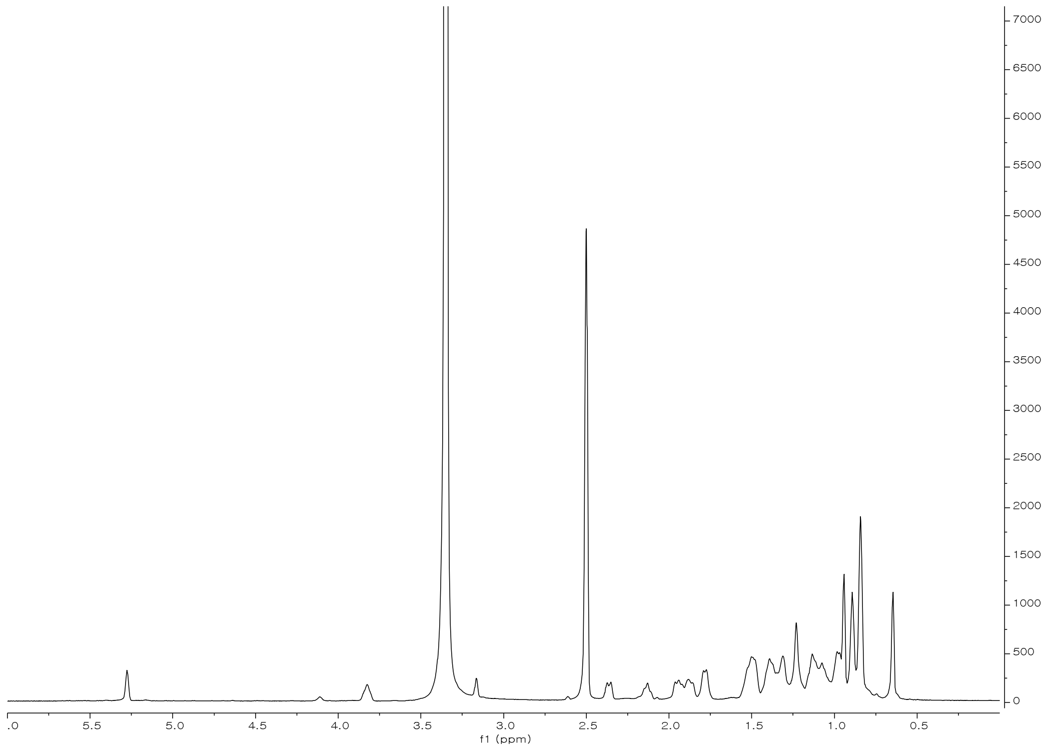

Figure S4. ^{1}H and ^{13}C NMR Spectra (500 MHz and 125 MHz, methanol-d_4) of **3**

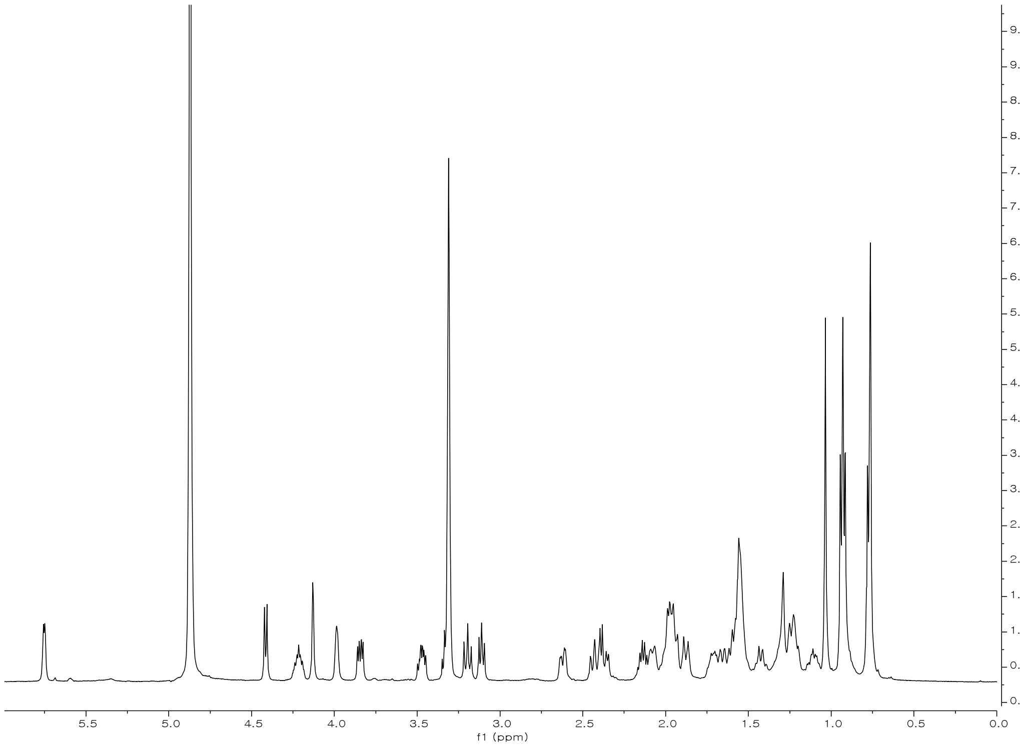

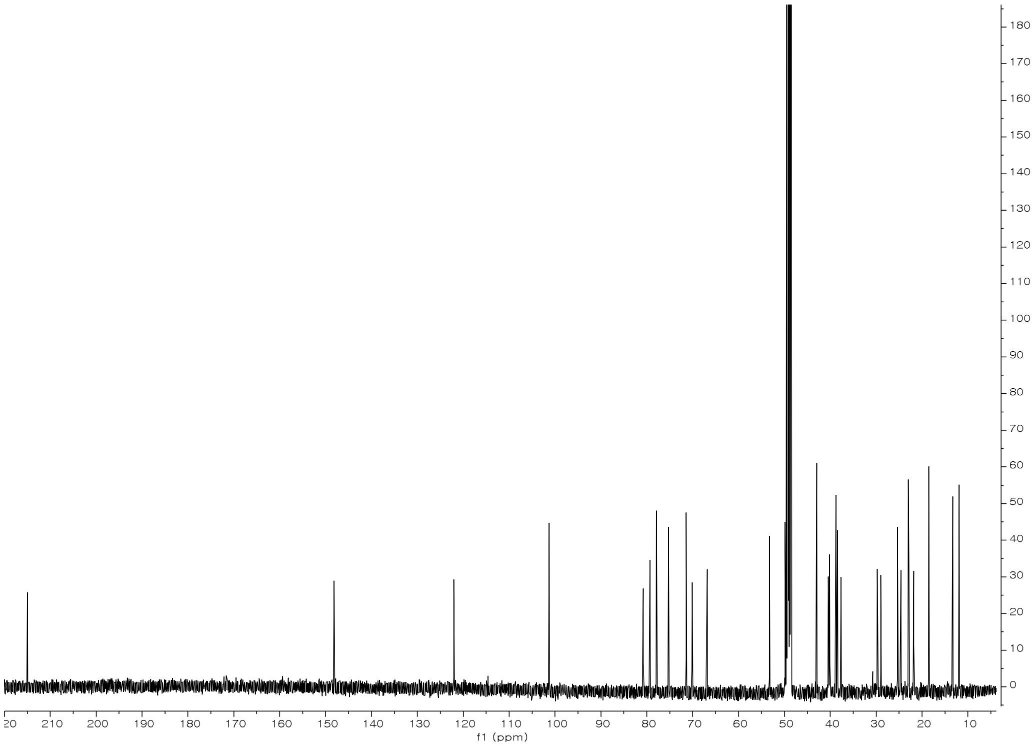

Figure S5. HMBC and HSQC NMR spectra of **3**

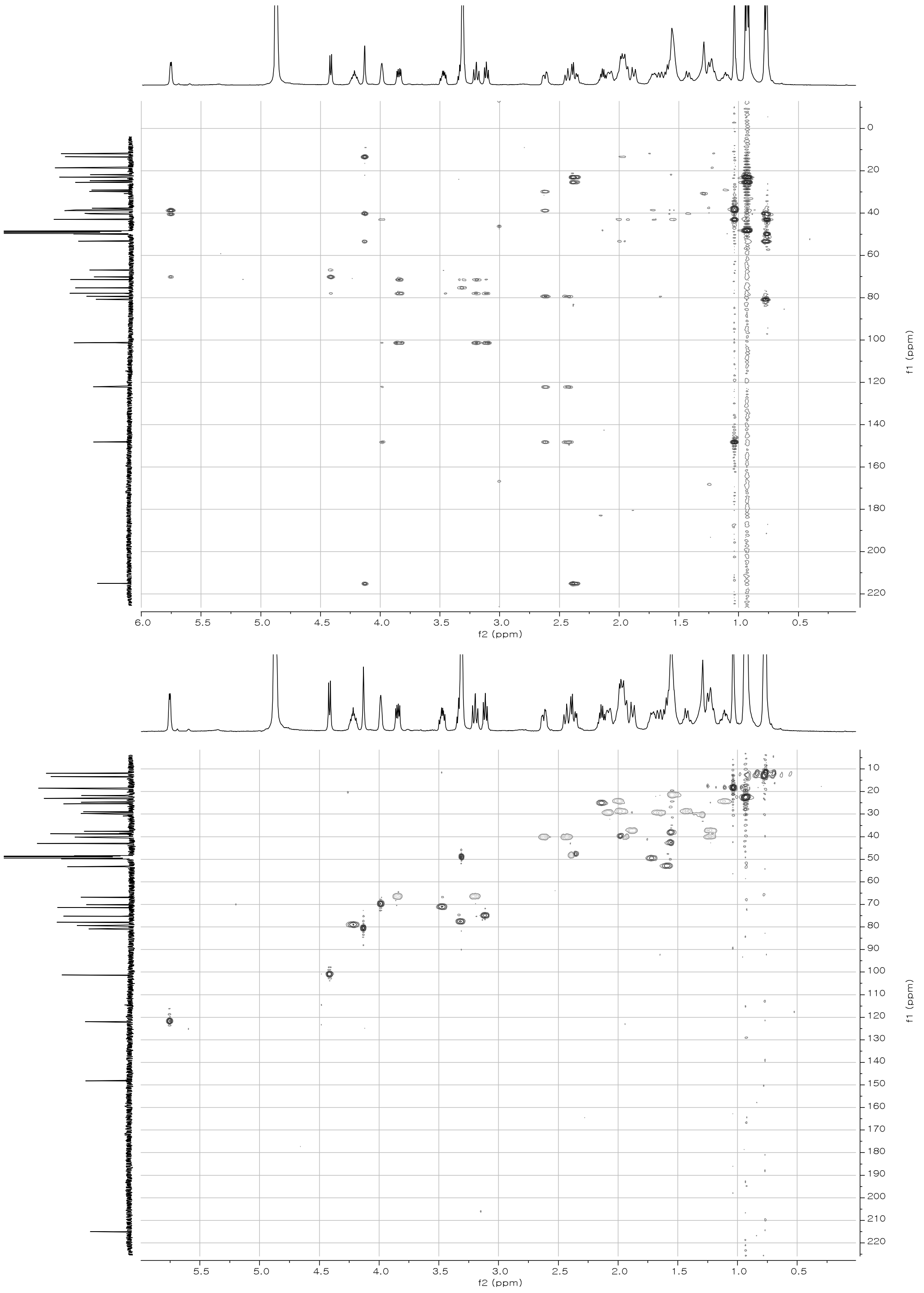

Figure S6. COSY and NOESY NMR spectra of **3**

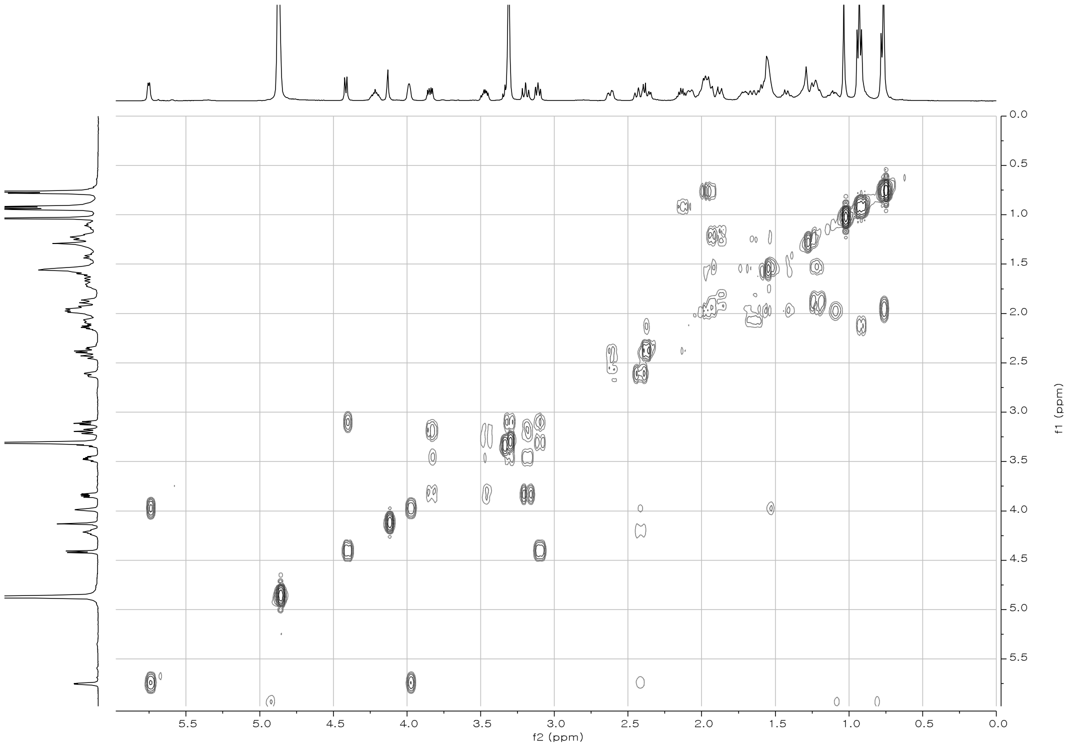

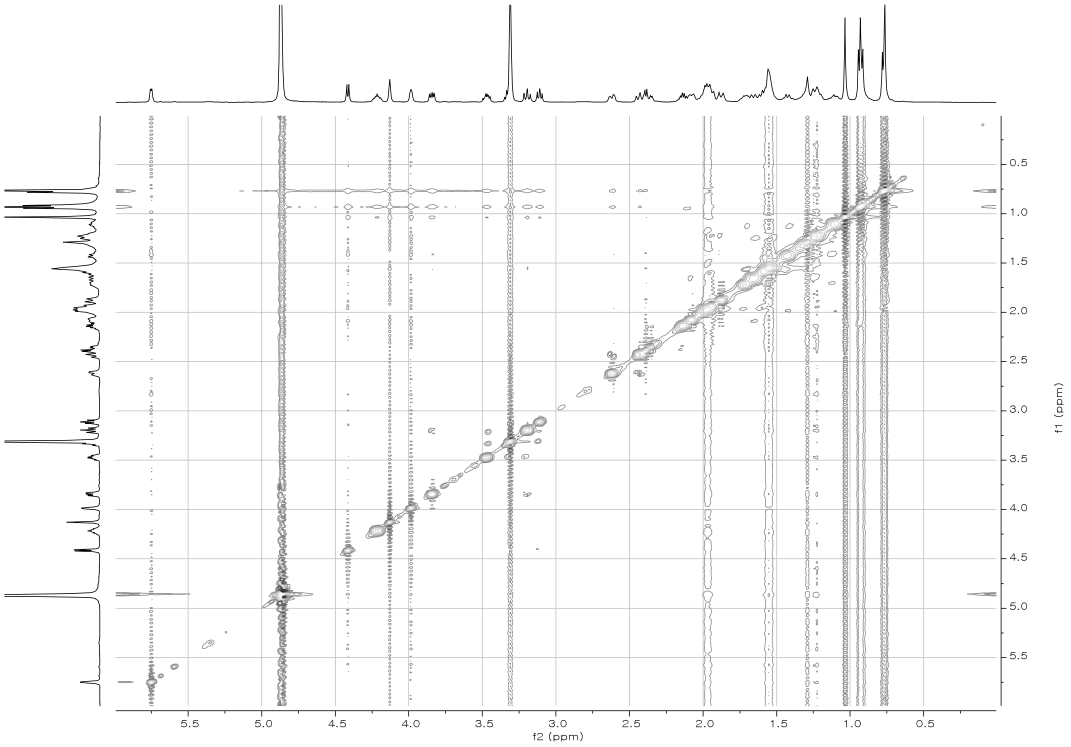

Figure S7. ^{1}H and ^{13}C NMR Spectra (500 MHz and 125 MHz, methanol-d_4) of **4**

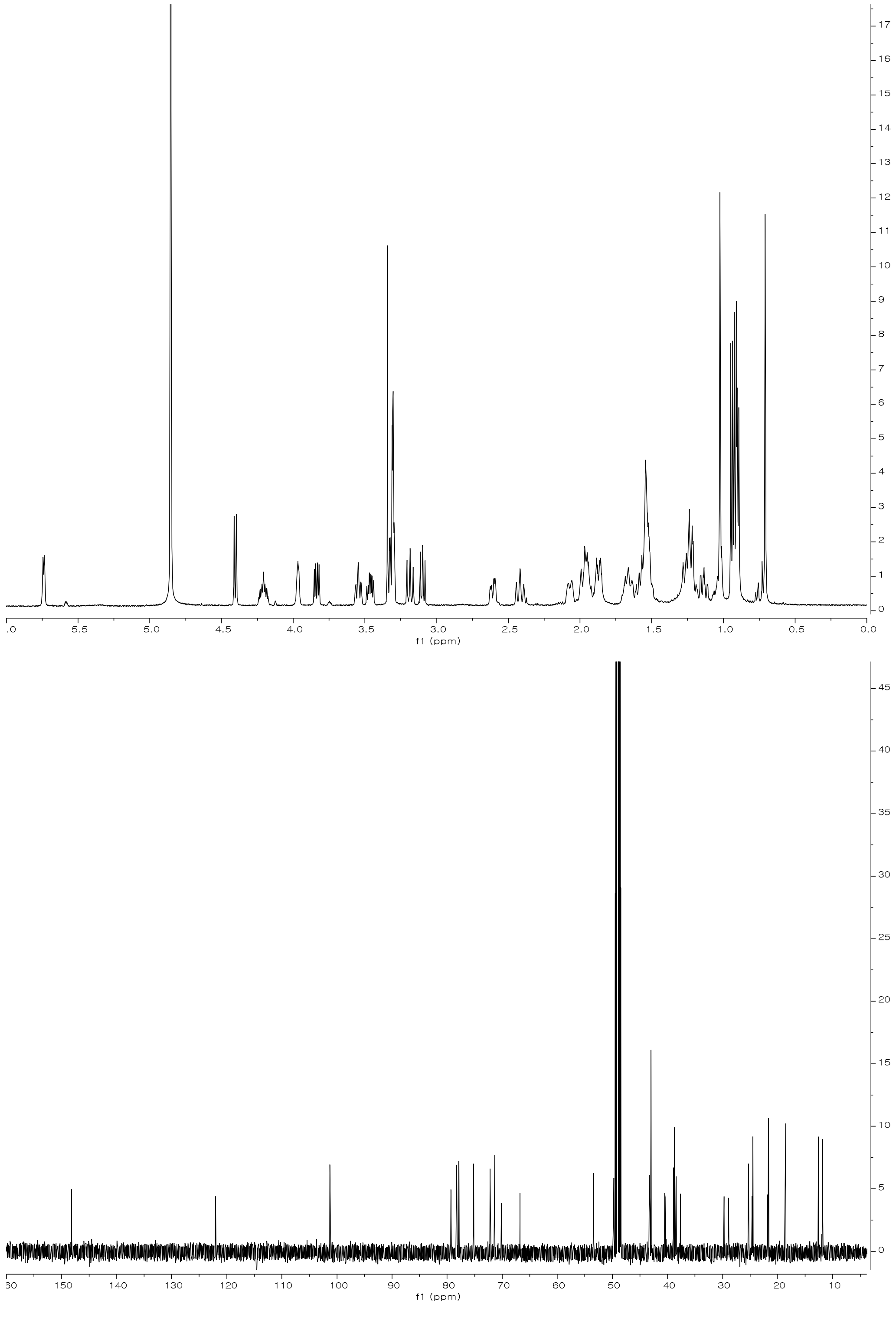

Figure S8. HMBC and HSQC NMR spectra of **4**

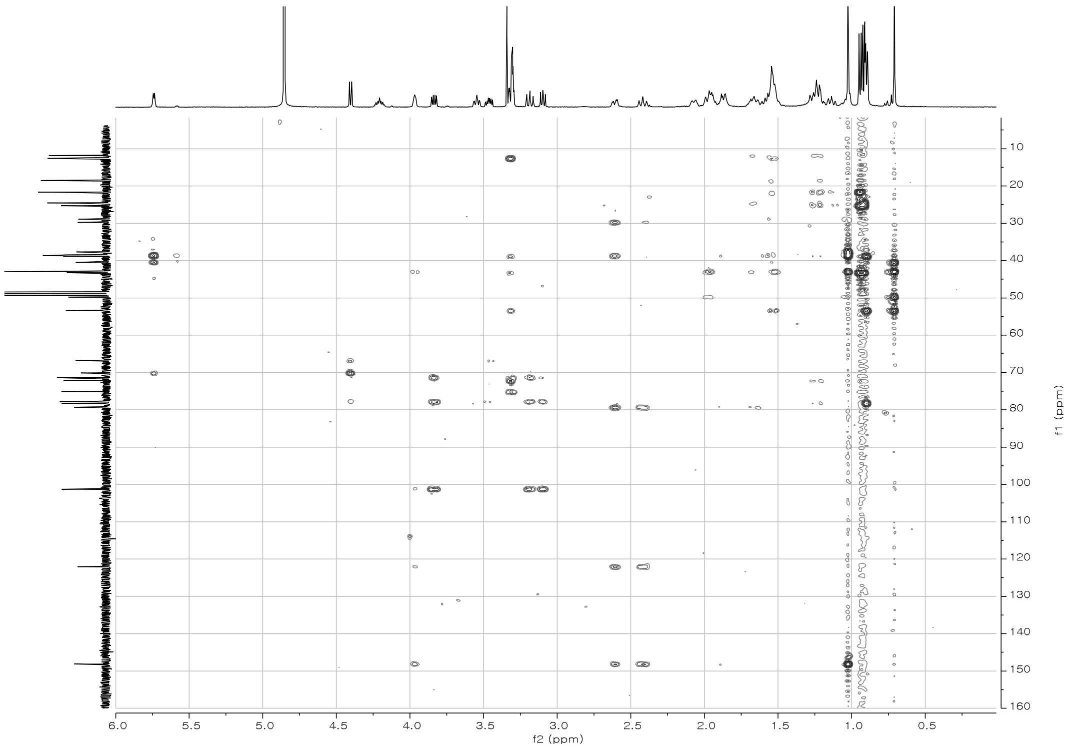

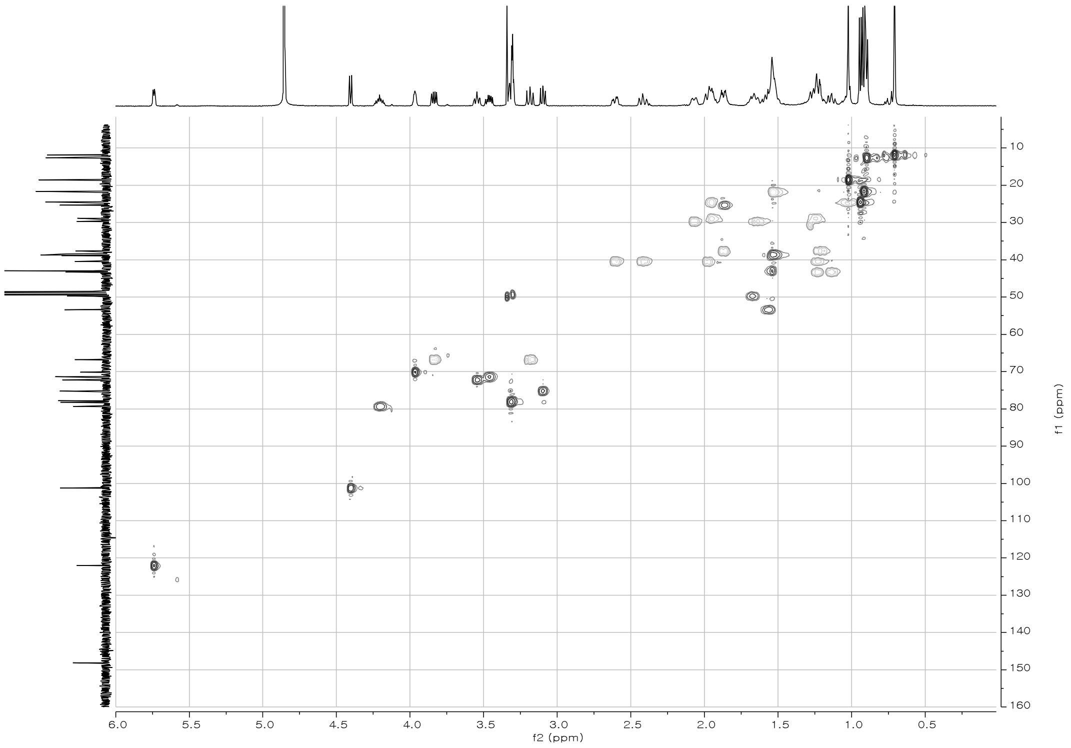

Figure S9. COSY and NOESY NMR spectra of **4**

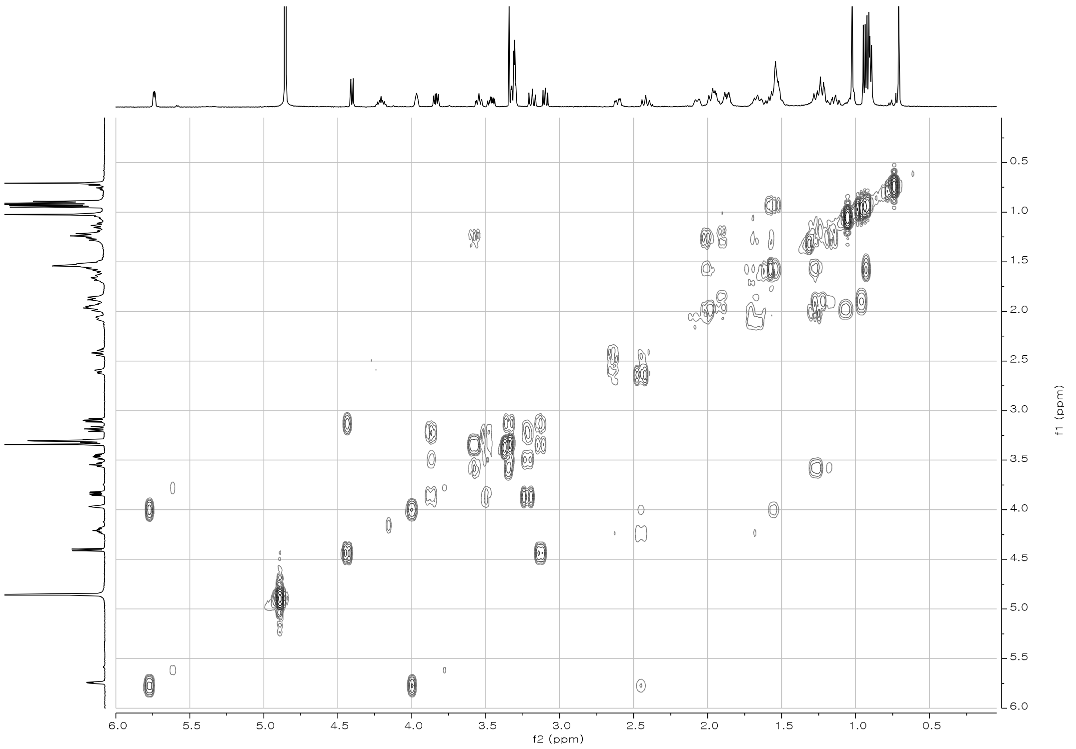

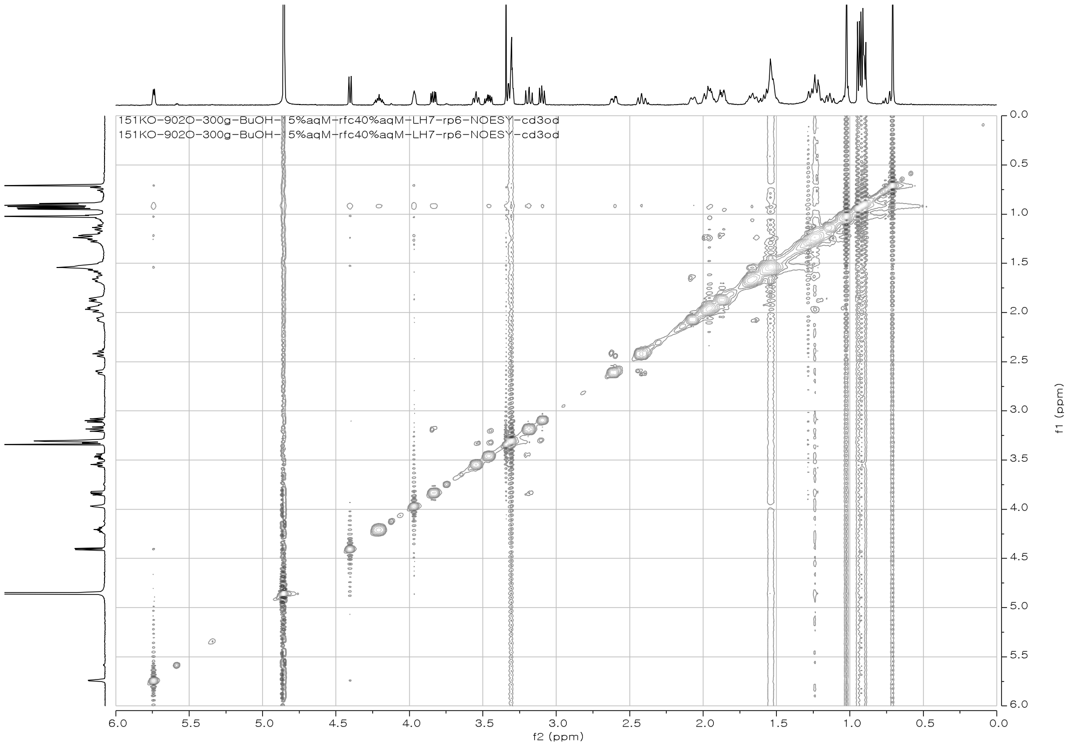

Figure S10. ^{1}H and ^{13}C NMR Spectra (500 MHz and 125 MHz, methanol-d_4) of **5**

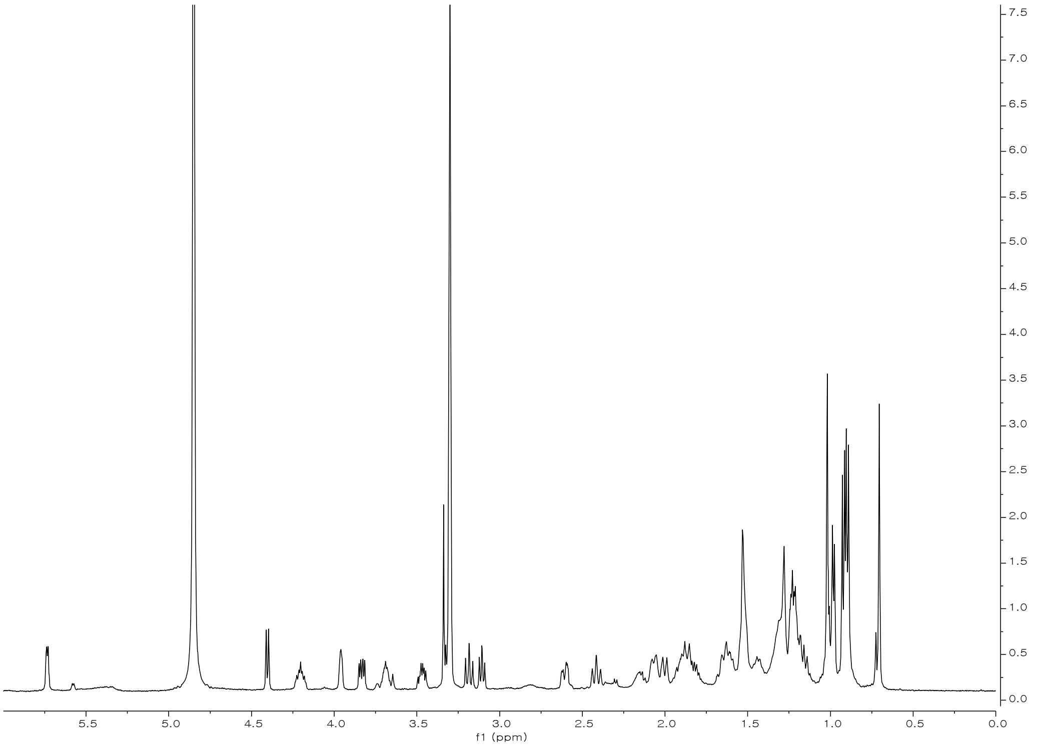

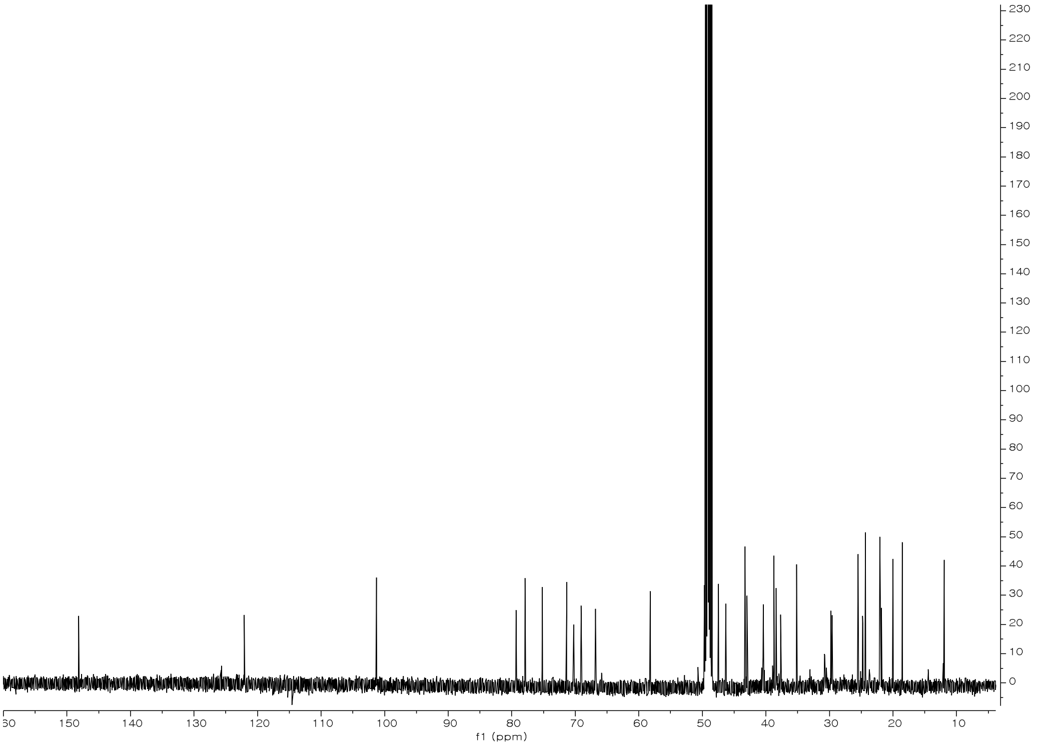

Figure S11. HMBC and HSQC NMR spectra of **5**

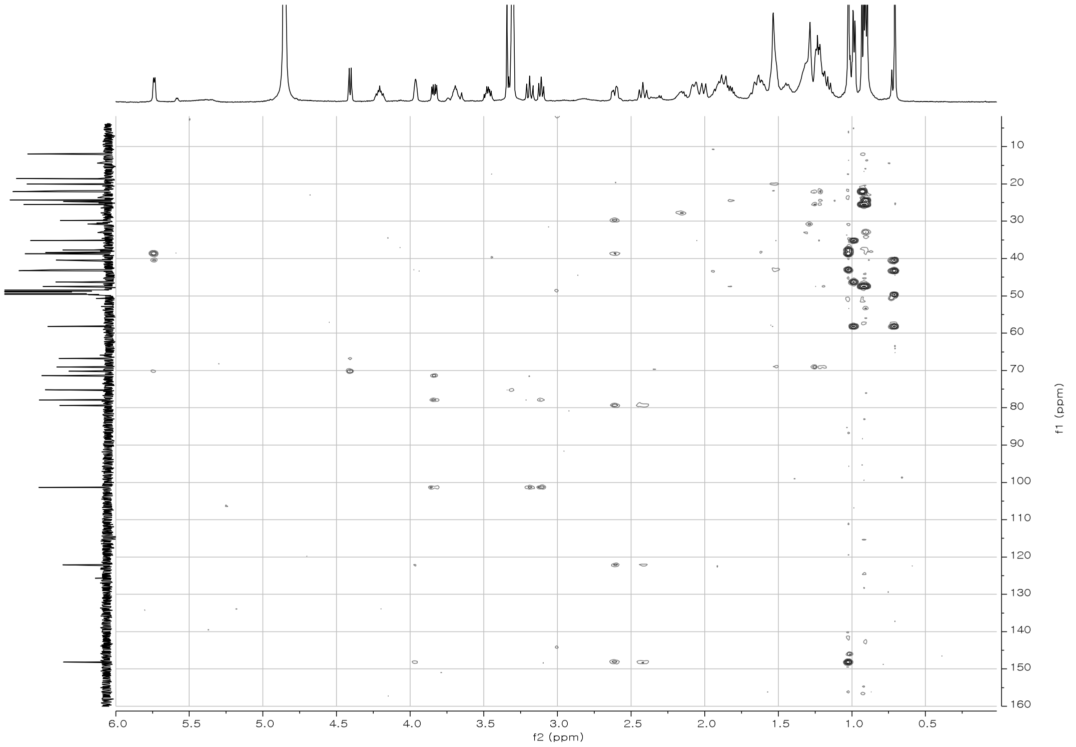

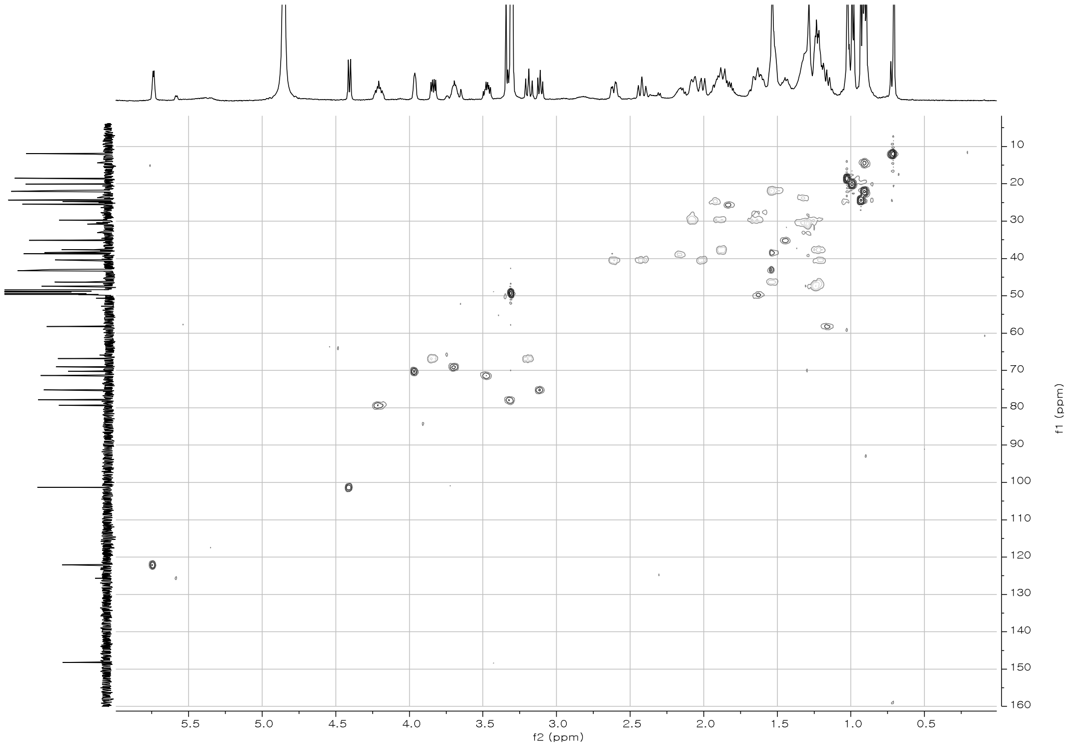

Figure S12. COSY and NOESY NMR spectra of **5**

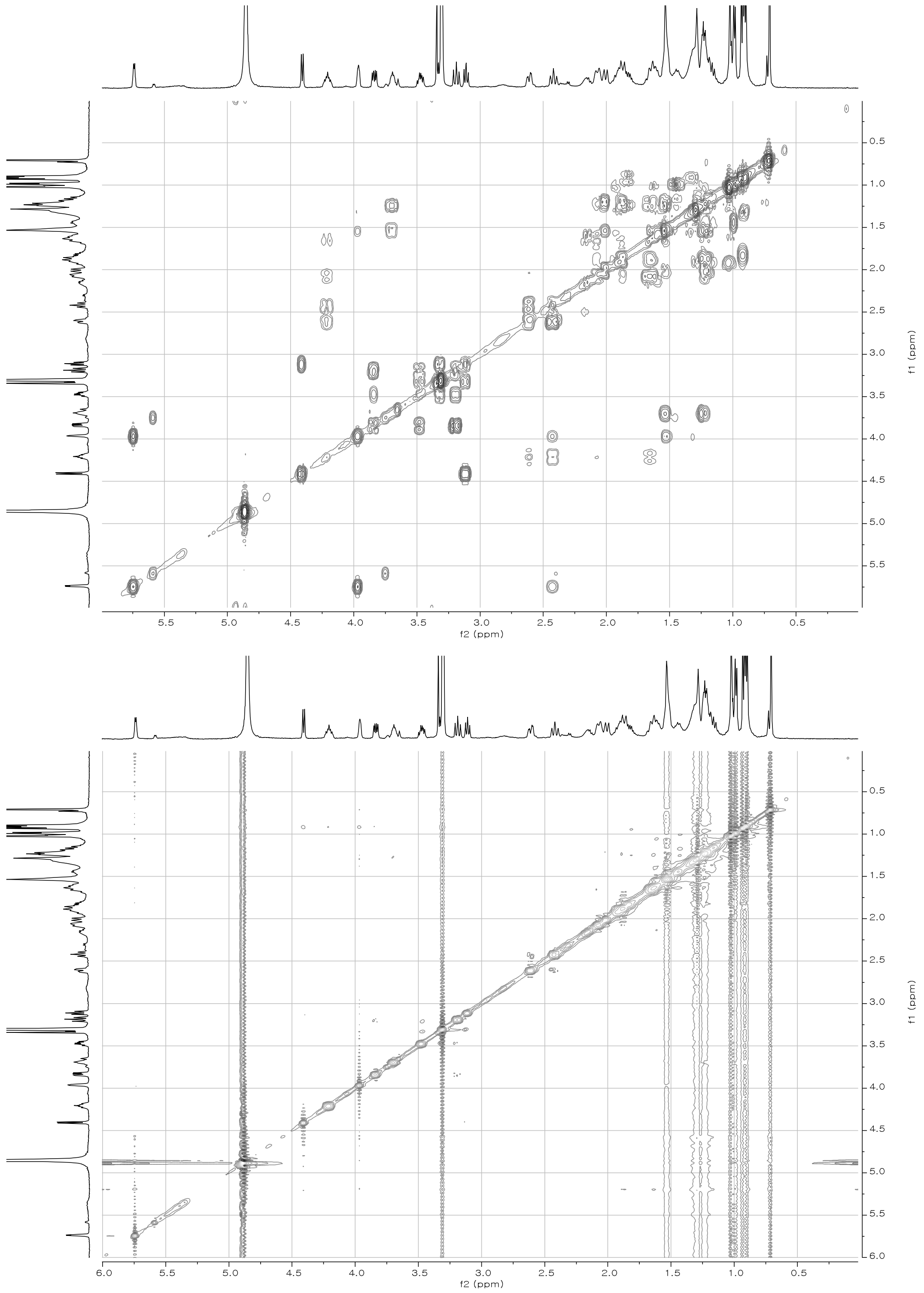

Supplement: Supplementary file 1 [file marinedrugs-15-00379-s001.pdf]
